# Evaluating Degradation at Railway Crossings Using Axle Box Acceleration Measurements

**DOI:** 10.3390/s17102236

**Published:** 2017-09-29

**Authors:** Zilong Wei, Alfredo Núñez, Zili Li, Rolf Dollevoet

**Affiliations:** Section of Railway Engineering, Faculty of Civil Engineering and Geosciences, Delft University of Technology, Stevinweg 1, 2628 CD Delft, The Netherlands; Z.Wei@tudelft.nl (Z.W.); A.A.NunezVicencio@tudelft.nl (A.N.); R.P.B.J.Dollevoet@tudelft.nl (R.D.)

**Keywords:** railway infrastructure monitoring, axle box acceleration measurement, crossing degradation, 3D profile measurement

## Abstract

In this paper, we investigate the capability of an axle box acceleration (ABA) system to evaluate the degradation at railway crossings. For this purpose, information from multiple sensors, namely, ABA signals, 3D rail profiles, Global Positioning System (GPS) and tachometer recordings, was collected from both nominal and degraded crossings. By proper correlation of the gathered data, an algorithm was proposed to distinguish the characteristic ABA related to the degradation and then to evaluate the health condition of crossings. The algorithm was then demonstrated on a crossing with an unknown degradation status, and its capability was verified via a 3D profile measurement. The results indicate that the ABA system is effective at monitoring two types of degradations. The first type is uneven deformation between the wing rail and crossing nose, corresponding to characteristic ABA frequencies of 230–350 and 460–650 Hz. The second type is local irregularity in the longitudinal slope of the crossing nose, corresponding to characteristic ABA frequencies of 460–650 Hz. The types and severity of the degradation can be evaluated by the spatial distribution and energy concentration of the characteristic frequencies of the ABA signals.

## 1. Introduction

**Figure 1:**
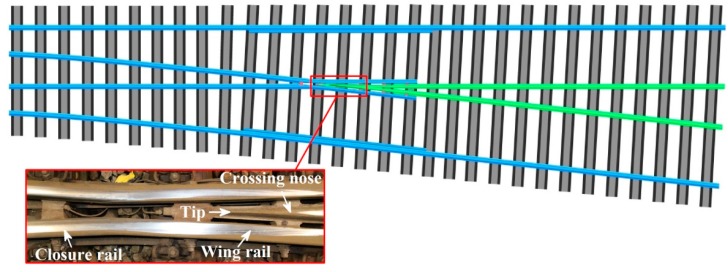
Schematic diagram of a railway crossing.

A railway crossing is one of the fundamental components of track infrastructure because it intersects different tracks at the same level. Figure 1 shows a schematic diagram of a crossing. A crossing constitutes a geometric discontinuity (i.e., a gap) between the closure rail and crossing nose such that the clearance of a wheel flange is guaranteed. During the passage of vehicles, high wheel-rail impact forces and undesirable vibrations arise and accelerate degradation at crossings. Crossings account for a large fraction of maintenance and renewal costs in railway systems worldwide. For example, faults associated with switches and crossings in Sweden correspond to over 13% of maintenance costs [1]. On the Dutch railway network, a total of 826 crossings were replaced between 2011 and 2015 [2]. The Dutch railway network authority (ProRail) stipulates that severely degraded crossings must be replaced within 24 h after they are detected, resulting in unpredictable traffic delays in the railway system.

**Figure 2:**
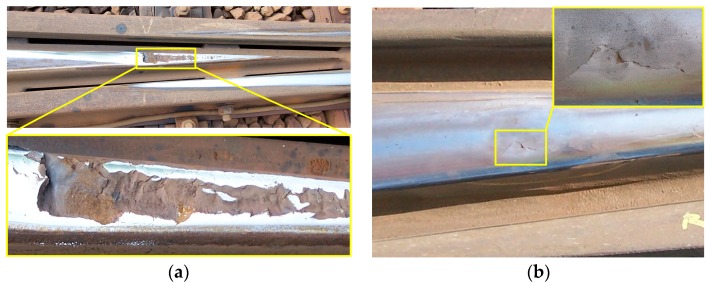
Examples of severe degradation at crossings. (**a**) Shelling: a subsurface fatigue phenomenon associated with high wheel-rail contact stresses; (**b**) squats: a local rail top deformation in the running band.

Figure 2 shows two examples of severe degradation at crossings. Both crossings required urgent replacement due to shelling and squats [3]. If these degradations were to be identified at an early stage when they are local and minimal, preventive maintenance (e.g., grinding and welding) could be performed, and their service life could be extended. Therefore, an effective diagnostic method is required for the prompt detection of degradations at crossings.

Currently, the monitoring of crossings highly relies on human visual inspection, that is, technicians visit the tracks or watch videos of tracks to identify degradation. This task is labor-intensive and inefficient, and the detection results may be subjective and erroneous. Consequently, most degraded crossings are reactively replaced when they reach severe degradation. The limitations of manual inspection motivate the development of on-board monitoring technologies, such as ultrasonic measurement [4,5], eddy current testing [6,7], magnetic induction [8], image recognition [9,10,11], vibration-based inspection [12,13], guided-wave inspection [14], radio detection and ranging sensors [15,16], thermography [17,18], acoustic emission systems [19,20] and ground-penetrating radar [21,22].

Each of these technologies is adept at detecting certain defects but suffers from certain limitations. Ultrasonic measurement shows the best performance in detecting cracks, whereas it is not adept at detecting surface detects. Eddy current testing is able to detect surface defects. However, the probe in the eddy current system must be positioned at a constant distance from the rail head, making it unavailable at geometric discontinued crossings. The magnetic induction method, which can be used for near-surface or surface transverse defects, also suffers from the restriction of a constant distance between sensors and rail surfaces. The image recognition method can detect visible defects on rail surface and other track components (e.g., loose bolts and moving sleepers), whereas it is adversely affected by illumination inequality and inconsistent reflection properties on plain tracks and by the complex structure and discontinued geometry at crossings. Vibration-based inspection can be used for several purposes, such as monitoring track irregularity and detecting train derailment, whereas some of its applications (such as detecting long wave rail irregularity) require moderate train speeds. Guided-wave inspection is capable of detecting several defect types, such as cracks and corrosion, but is limited by the critical size of defects. Radio detection and ranging sensors are able to detect obstacles endangering railway operation (e.g., moving cars at level crossings). Acoustic emission systems can detect several defects, such as poor track alignment and squats, but its on-board implementation is disturbed by the noise generated by airflows. The thermography method can detect cracks and ballast conditions, but the method is disturbed by fluctuations in weather conditions. Ground-penetrating radar is adept at monitoring track substructure components such as ballasts and subgrades. Among the abovementioned inspection methods, those that can be applied at geometric discontinued crossings (e.g., ultrasonic measurement) are adept at crack detection. Regarding the numerous crossings without cracks yet with significant profile degradation because of plastic deformation and wear, an effective diagnostic method is required for prompt detection.

In this study, we investigate the feasibility of the axle box acceleration (ABA) system for the condition monitoring of railway crossings. ABA measurement is a vibration-based inspection method, and its detection algorithm relies on extracting changes in dynamic responses with respect to the nominal condition. On plain tracks, the ABA system has been used to detect isolated defects (e.g., squats [23], poorly qualified welds [24] and worn insulated rail joints [25]) and periodic defects (e.g., corrugation [26,27,28] and wheel flats [29]).

At railway crossings, the application of the ABA system is more complex because of the complex structure and discontinued geometry. The ABA signals measured at crossings contain information related to both the inherent geometric discontinuity and the undesired defects; thus, the ABA system must be able to identify the characteristic vibrations related to defects [30]. Few studies in the literature have applied an ABA system to crossings [31,32]. These studies did not distinguish the dynamic responses belonging to different sources, making them unable to evaluate the type, location and severity of crossing defects.

**Figure 3:**
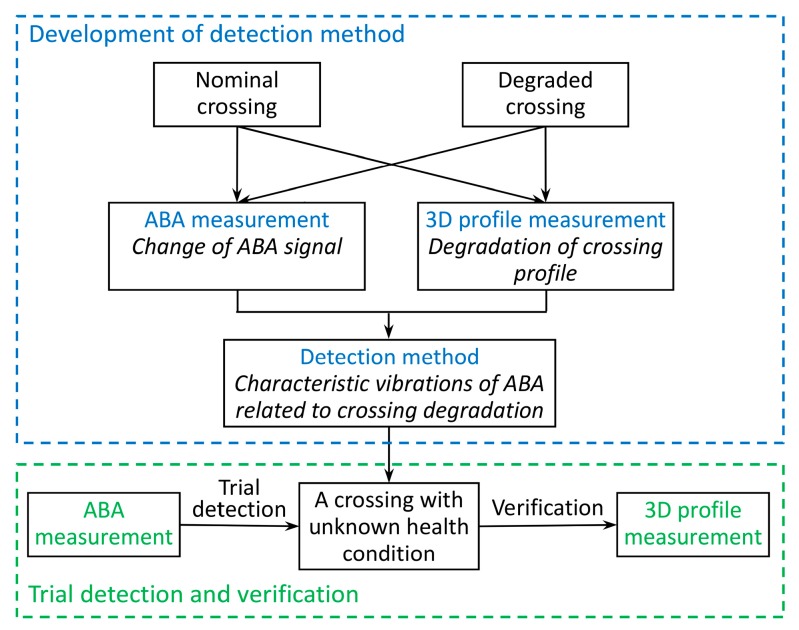
Structure of the methodology.

To overcome the challenges faced in these previous works, this study measures both ABA signals and crossing profiles over time and attempts to extract the relationships between the gathered data. With the aid of an interpretation-based approach, the ABA system is potential to estimate the type, location and severity of crossing defects. Figure 3 shows the structure of the methodology. Information from multiple sensors, namely, ABA signals, Global Positioning System (GPS), tachometer recordings and measured 3D profiles, was obtained from reference crossings of the same type with different conditions (both nominal and degraded) to extract the signature vibrations related to crossing degradation.

The paper is organized as follows. Section 2 presents the ABA and 3D profile measurement systems. Section 3 investigates the characteristic vibrations of ABA related to the degradation at a crossing and proposes a detection method. In Section 4, ABA measurement is performed on a crossing with an unknown degradation status as a trial, and the detection results are verified using 3D profile measurements and field observations. Finally, Section 6 presents conclusions and recommends possible future work.

## 2. ABA and 3D Profile Measurements

In this section, in situ ABA and 3D profile measurements were conducted on two reference crossings. The two crossings are of the same type: a 54E1-1:9 type with a UIC54 rail profile and a crossing angle of 1:9. The crossings have different profiles: the first was newly installed and nearly in the nominal state, while the second had been in the track for 12 years and had degraded significantly. At both crossings, the train operational speed was limited to 40 km/h, and over 96% of the total traffic loads (approximately 20 million gross tons per year) occurred in the facing direction, namely, from closure rail to crossing nose.

### 2.1. ABA Measurement

**Figure 4:**
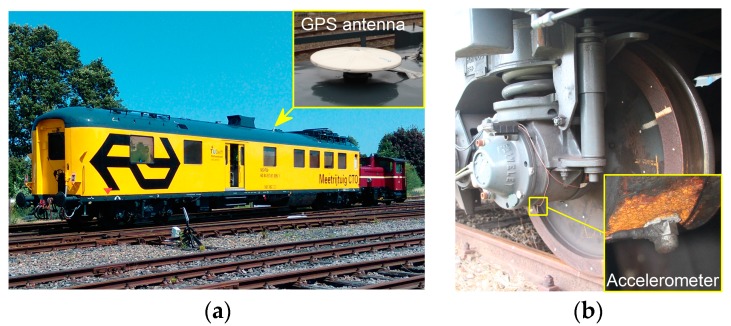
(**a**) Axle box acceleration (ABA) measuring train and (**b**) accelerometer configuration.

The ABA system is composed of accelerometers, a GPS antenna and a tachometer; see Figure 4. The accelerometers are mounted on the axle boxes of the train to measure the vertical ABA signals. The sampling frequency of ABA is set to 25.6 kHz to ensure that sufficient information is captured from the moving sensors over a broad range of measuring speeds. The GPS antenna is installed on the train roof to record the location, and the tachometer is used to record train speed and ensure optimal positioning of the ABA signals.

**Figure 5:**
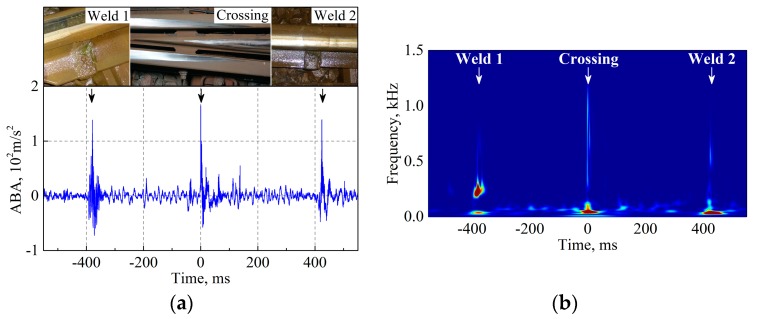

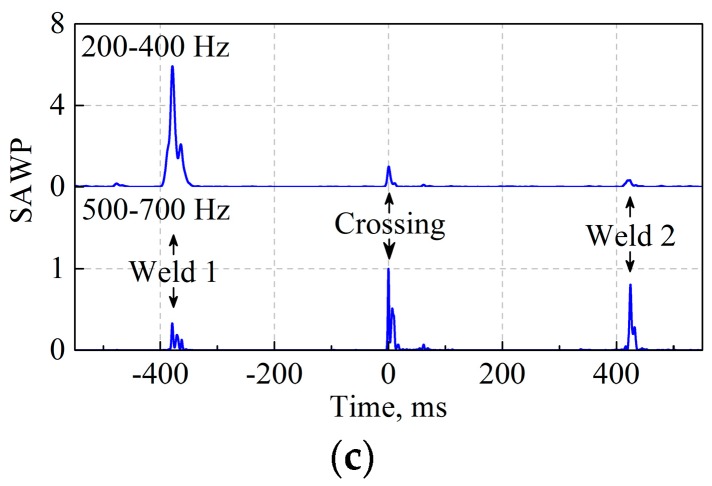
An example of the measured ABA: (**a**) time history; (**b**) wavelet power spectrum (WPS), and; (**c**) scale-averaged wavelet power (SAWP).

When a train passes over ordinary tracks under nominal conditions, the ABA signal fluctuates because of the natural response of the vehicle-track system. The presence of undesired degradation (e.g., poor qualified welds and worn rail pads) or intrinsic geometric discontinuity (e.g., crossing and insulated rail joint) can significantly affect the vehicle-track interaction, which is captured in the ABA signal. Figure 5a shows an example of the measured ABA at a crossing and two adjacent welds. At the crossing and welds, only a minimal difference arises in the amplitudes and durations of the ABA signals; thus, it is not straightforward to first differentiate them and then extract sufficient information to evaluate their degradation condition in the time domain. Consequently, frequency analysis is applied for degradation detection because it can provide crucial information that is challenging to detect in the time domain.

The continuous wavelet transform (CWT) method has been effectively used to identify the characteristic vibrations of ABA at squats and worn insulated joints [25,32]. With the adjustment of certain parameters, the method can also be used to detect degradation at crossings. Wavelet analysis is used because the signal processing is independent of the window size, making it suitable for investigating the transient processes of brief events [33]. In the CWT, the convolutions of the analyzed signal are calculated with a group of scaled and shifted wavelet functions. The wavelet coefficients $W_{n}\left(s\right)$ of the analyzed signal x can be represented as follows [34]:

(1) $$W_{n}\left(s\right)=\sum_{n^{\prime }=0}^{N-1}x_{n^{\prime }}\psi^{*}\left[\frac{\left(n^{\prime }-n\right)\delta_{t}}{s}\right]$$

where ψ is the mother wavelet, s is the wavelet scale, N is the number of points in the time series, $n^{\prime }=0,\ldots ,N-1$, $\delta_{t}$ is the time step, n is the continuous variable for the translation, ∗ denotes a complex conjugate and $\psi^{*}\left[\frac{\left(n^{\prime }-n\right)\delta_{t}}{s}\right]$ is a family of wavelets deduced from the mother wavelet by various translation and scaling steps. Here, the Morlet function is employed as the mother wavelet [35].

Figure 5b is an example of the wavelet power spectrum (WPS), which is calculated using the square of the wavelet coefficients $\left|W_{n}^{2}\left(s\right)\right|$. The color indicates the amount of energy concentrated at a certain position (the horizontal axis) and frequency (the vertical axis). In the figure, the crossing and welds correspond to different characteristic frequencies and the energy concentration. To achieve improved quantification of the distributions of the WPS in certain frequency bands, the scale-averaged wavelet power (SAWP) is calculated. The SAWP is defined as the weighted sum of the WPS in the frequency band $f_{1}$ to $f_{2}$ [36]:

(2) $${\bar{W}}_{n}^{2}\left[f_{1},f_{2}\right]=\frac{\delta_{j}\delta_{t}}{C_{\delta }}\sum_{j=f_{1}}^{f_{2}}\frac{{\left|W_{n}\left(s_{j}\right)\right|}^{2}}{s_{j}}$$

where $\delta_{j}$ is the scale step and $C_{\delta }$ is an empirically derived reconstruction factor. To facilitate the comparison of different scenarios, the SAWP is divided by a constant γ:

(3) $${\bar{W}}^{2}\left[f_{1},f_{2}\right]=\frac{{\bar{W}}_{n}^{2}\left[f_{1},f_{2}\right]}{\gamma }$$

where γ is the value of the SAWP in the nominal state and at the position of wheel-crossing impact.

Figure 5c shows an example of the SAWP at 200–400 Hz and 500–700 Hz. The two welds correspond to different frequency responses: weld 1 exhibits a large value of ${\bar{W}}^{2}\left[200,400\right]$ and a small value of ${\bar{W}}^{2}\left[500,700\right]$, in contrast with the values at weld 2. This information reveals that the two welds feature different degradation conditions. The crossing health condition can also be evaluated by comparing the SAWP of the measured ABA with the value in the nominal state.

### 2.2. 3D Profile Measurement

**Figure 6:**
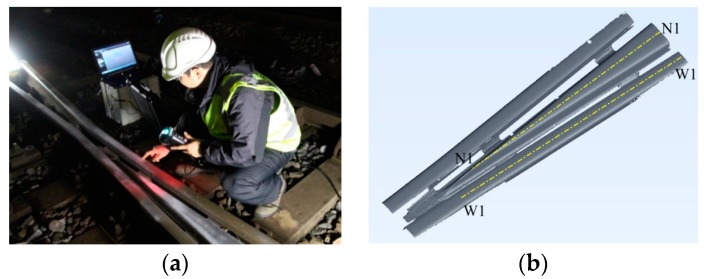
3D profile measurement. (**a**) Measurement using HandyScan and (**b**) a measured 3D crossing profile. Cross sections W1 and N1 are along the centerlines of the wing rail and crossing nose, respectively.

The crossing profile was measured in 3D using the laser-based non-contact apparatus HandyScan; see Figure 6a. In this measurement, the laser stripes project a reference on the rail surface, which is captured by two cameras. The accuracy of HandyScan is 0.03 mm in arbitrary directions. Figure 6b shows an example of the measured 3D profile.

**Figure 7:**
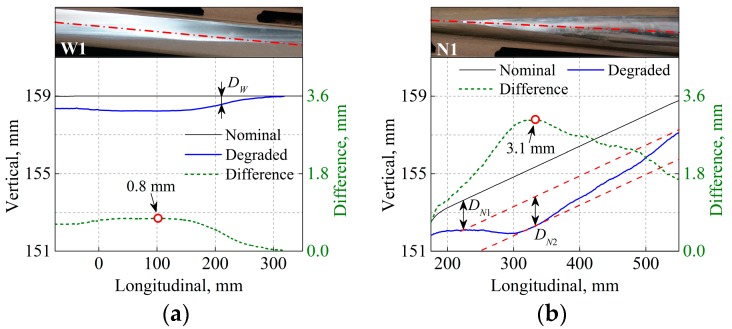
Measured crossing profile along cross sections W1 and N1. (**a**) Wing rail and (**b**) crossing nose. The dot (○) indicates the maximum difference in height between the nominal and degraded states. $D_{W}$ denotes the difference in the height of the wing rail, while $D_{N1}$ and $D_{N2}$ denote the difference in the height of the crossing nose.

To gain insight into the measured profile, two longitudinal-vertical cross sections are selected from the wing rail and crossing nose; see Figure 6b. The cross sections W1 and N1 are along the centerlines of the wing rail and crossing nose, respectively. Figure 7 compares the crossing profile of the nominal and degraded states, where the origin of the abscissa aligns with the tip of the crossing nose. Along W1, the height difference between the nominal and the degraded profiles reaches the maximum of 0.8 mm at 102 mm. Because of the misalignment of the rolling direction of the wheelset and the centerline of the wing rail, the running band on the wing rail shrinks laterally towards the gauge side, ultimately reaching zero at 305 mm.

On the crossing nose, wheel-rail contact occurs from 235 mm onward, as indicated by the shiny running band in Figure 7b. In this region, the difference in height between the nominal and degraded state is $D_{N1}$, which is larger than the difference in the height of the wing rail $D_{W}$ between the two states. The difference increases along N1 and reaches its maximum of 3.1 mm at 333 mm. Thereafter, the height difference decreases such that the degradation at $D_{N2}$ is more severe than in its surrounding area, leading to local irregularity in the longitudinal slope of the crossing nose.

**Figure 8:**
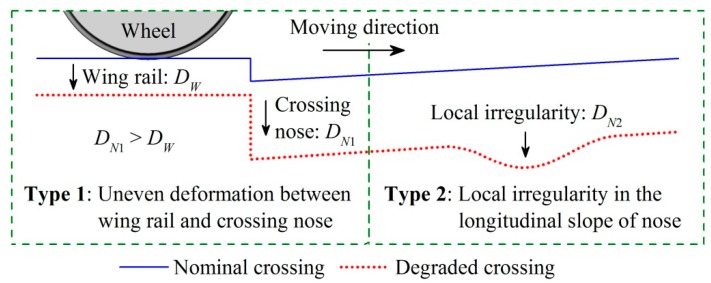
Two types of degradation at the measured crossing. $D_{W}$ denotes the difference in the height of the wing rail, while $D_{N1}$ and $D_{N2}$ denote the difference in the height of the crossing nose.

Based on the measured crossing profiles shown in Figure 7, the degradation at the measured crossing can be divided into two types, as illustrated in Figure 8. The first type is the uneven deformation between the wing rail and the crossing nose (i.e., $D_{N1}>D_{W}$ in Figure 7), which is caused by the dominance of the facing motion of vehicles and the related large impact forces and stresses on the crossing nose. The second type is the local irregularity in the longitudinal slope of the crossing nose (i.e., $D_{N2}$ in Figure 7b). This local irregularity is caused by the complex crossing geometry and the related non-identical distributions of the plastic deformation and wear on the crossing nose [37].

## 3. Characteristics of ABA Related to Degradation at Crossing

The characteristic vibrations of ABA related to the degradation are extracted by comparing the measured ABA and 3D profiles of nominal and degraded crossings. With this information, an algorithm capable of detecting crossing degradation is proposed.

### 3.1. Repeatability of Measured ABA

To demonstrate the repeatability of the measured ABA, the measurements were performed three times using the same train at the nominal crossing. In each measurement, the train passed over the crossing at a speed of 26–28 km/h in the facing direction.

**Figure 9:**
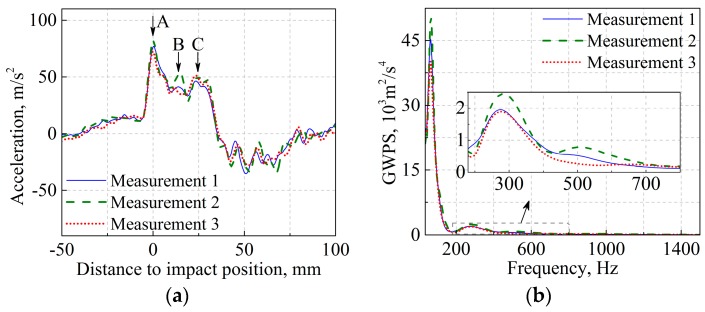
Measured ABA at the nominal crossing: (**a**) time history, and; (**b**) global wavelet power spectra (GWPS).

Figure 9a compares the measured ABA signals, where the origin of the abscissa is aligned with the crest of the first major peak, as indicated by arrow A. In general, these ABA signals coincide well with each other in terms of the waveform and amplitude of major peaks. There are three major peaks in all the signals, denoted by arrows A, B and C. The dispersions in the amplitudes at arrows A, B and C are up to 12%, 23% and 10% among the measurements. The dispersion is mainly attributed to the randomness of vehicle-track interaction, for example, the hunting oscillation. Relative to the amplitudes, the wavelengths of the three peaks are in closer agreement among the measurements. Because the wavelengths are relevant to the frequency components of ABA, the frequency components are preferred for evaluating crossing degradation.

Figure 9b compares the global wavelet power spectra (GWPS) of ABA, defined as the WPS averaged over the location at each frequency [36]. In the figure, the time history of ABA distributed between −28 and 28 ms is analyzed to reduce the disturbance of vibrations far from the wheel-rail impact. In general, satisfactory agreement can be achieved in terms of the major frequency components. The most dominant frequency occurs at approximately 65 Hz, while higher-frequency components of approximately 270 and 520 Hz can also be observed, albeit with much lower energy. High vibration energy is concentrated in the low-frequency range for the following two reasons. First, the energy of the vehicle-track interaction depends on the train speed, particularly in the high-frequency range [38]. In the measurements, a low measuring speed (i.e., 26–28 km/h) makes the high-frequency response less pronounced than the components of the low-frequency response. Second, the high-frequency components, which are generally localized within short durations, are averaged over a long duration from −28 to 28 ms, thus reducing the magnitude of the GWPS.

In Figure 9, the measured ABA signals are coherent with each other in terms of the amplitudes of the major peaks and the major frequency components. Because of the non-identical moving trajectory of the wheelset, it is challenging to achieve a precise match between measurements. Nevertheless, the ABA measurement demonstrates satisfactory repeatability in both the spatial and frequency domains, which is essential for detecting crossing degradation.

### 3.2. Comparison of Dynamic Response between Nominal and Degraded Crossings

**Figure 10:**
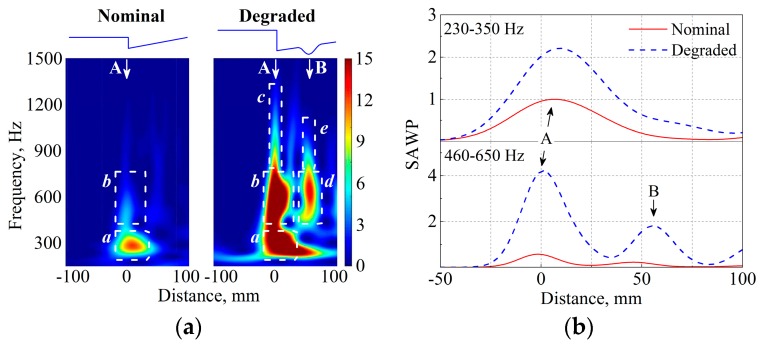
Comparison of the ABA at nominal and degraded states. (**a**) WPS and (**b**) SAWP. The rectangles indicate the major characteristics. Arrows A and B indicate the location of degradation types 1 and 2.

Figure 10a compares the WPS of the nominal and the degraded states. In the figure, the frequency range is limited to 150–1500 Hz because of the following factors. First, the low-frequency components below 150 Hz mainly correspond to the properties of the track substructure components (such as the subgrade, ballast and sleeper) rather than the crossing rails [39]. Second, the high-frequency components above 1500 Hz have much lower energy than the lower-frequency components and are hardly visible on the same scale (the color bar). Figure 10b shows the SAWP, that is, ${\bar{W}}_{Nom}^{2}$ of the nominal state and ${\bar{W}}_{Deg}^{2}$ of the degraded state. The SAWP is calculated using the values ${\bar{W}}_{n}^{2}\left[230,350\right]$ and ${\bar{W}}_{n}^{2}\left[460,650\right]$ divided by the constant ${\bar{W}}_{Nom}^{2}\left[230,350\right]$ at the wheel-rail impact position.

In the nominal state, most of the vibration energy is localized around arrow A, which is induced by the intrinsic geometric discontinuity of the crossing, and thus the wheel-rail impact. At the arrow, two major characteristic frequencies appear at 230–350 and 460–650 Hz; see rectangles *a* and *b* in Figure 10a.

In the degraded state, the vibration energy distributes at two regions, as denoted by arrows A and B. At arrow A, the major frequencies occur at 230–350 and 460–650 Hz. Because these characteristic frequencies take place in both nominal and degraded states, they should depend on the natural responses of the system rather than the wheel-rail contact geometry. The high-frequency components at arrow A (denoted as rectangle *c*) localize within a short duration such that both the train bogie and the track superstructure components are only minimally affected [40,41]. Therefore, the high-frequency response is expected to be relevant to the local vibration of contact bodies. The uneven deformation at arrow A (degradation type 1) increases the energy at the two characteristic frequencies. For example, the values of ${\bar{W}}_{Deg}^{2}\left[230,350\right]$ and ${\bar{W}}_{Deg}^{2}\left[460,650\right]$ increase by 121% and 631% compared to the values in the nominal state.

At arrow B, the value of ${\bar{W}}_{Deg}^{2}\left[460,650\right]$ reaches 1.8. The high vibration energy is related to local irregularity of the crossing nose (degradation type 2). The 460–650 Hz components occur at both degradation types 1 and 2 (arrows A and B), indicating that they are more related to the natural response of the system rather than to the wheel-rail contact geometry. The very-high-frequency components at rectangle *e* in Figure 10a, analogous to those in rectangle *c*, should originate from the local vibration of contact bodies.

By comparing the gathered data in the nominal and degraded states, the following relation between the characteristics of ABA (Figure 10) and the measured degradation (Figure 7 and Figure 8) can be extracted:

- Degradation type 1 (uneven deformation between the wing rail and the crossing nose). It exacerbates the wheel-rail impact and enlarging the energy concentrated at the characteristic frequencies of 230–350 and 460–650 Hz. The severity of the degradation can be evaluated by the values of ${\bar{W}}^{2}\left[230,350\right]$ and ${\bar{W}}^{2}\left[460,650\right]$ at the wheel-rail impact.
- Degradation type 2 (local irregularity in the longitudinal slope of the crossing nose). It increases the vibration energy at 460–650 Hz. Thus, the location of the irregularity can be determined by the spatial distribution of the 460–650 Hz components, while the severity can be evaluated by the value of ${\bar{W}}^{2}\left[460,650\right]$.

Figure 10a (rectangles *c* and *e*) shows that the high-frequency components at 650–1200 Hz become more pronounced in both degradation types. Because these components distribute with relatively short duration, they can be used to better locate the crossing degradation.

### 3.3. Detection Algorithm of Crossing Degradation

By proper correlation of the gathered data from multiple sensors, an algorithm for the detection of crossing degradation is proposed. In the algorithm, the SAWP is calculated using the values ${\bar{W}}_{n}^{2}\left[230,350\right]$ and ${\bar{W}}_{n}^{2}\left[460,650\right]$ divided by the value ${\bar{W}}_{Nom}^{2}\left[230,350\right]$ at the wheel-crossing impact position. The detection procedure is as follows:

**Step 1**: Detection of uneven deformation between the wing rail and the crossing nose (i.e., the first degradation type).

- If ${\bar{W}}^{2}\left[230,350\right]\geq 1$ and ${\bar{W}}^{2}\left[460,650\right]\geq 0.6$, then the crossing suffers from uneven deformation. Its severity increases with the increase of ${\bar{W}}^{2}\left[230,350\right]$ and ${\bar{W}}^{2}\left[460,650\right]$.
- Otherwise, the crossing does not exhibit significant uneven deformation.

**Step 2**: Detection of local irregularity of the crossing nose (i.e., the second degradation type).

- If there is more than one position with ${\bar{W}}^{2}\left[460,650\right]\geq 0.3$, then the crossing suffers from irregularity at the nose. Its severity increases with the increase in ${\bar{W}}^{2}\left[460,650\right]$.
- Otherwise, the crossing does not exhibit significant local irregularity at the nose.

## 4. Case Study: Trial Detection and Verification

In this section, the ABA measurement is performed on a crossing with unknown degradation status as a trial detection, and the capability of the proposed detection algorithm is verified via in situ profile measurement and field observation.

### 4.1. The Crossing with Unknown Degradation Status

**Figure 11:**
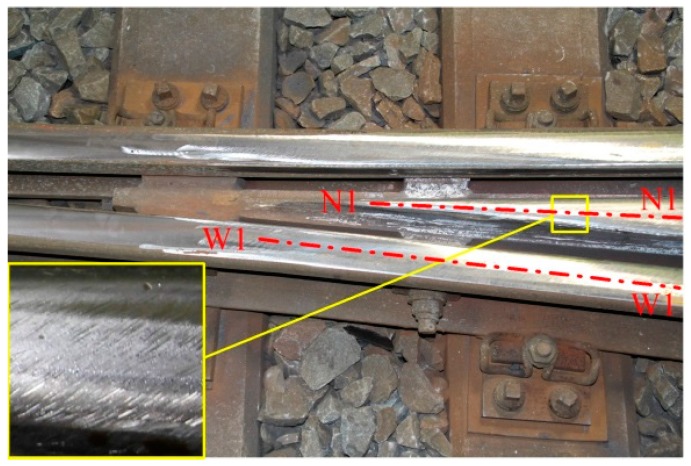
Repaired crossing profile with a magnified image of grinding marks. The degraded crossing was repaired through a grinding-welding-grinding process: first, rail surface damages were removed via grinding; second, hollow regions were filled by welding material; last, the rails were ground again to shape the profile.

Because the concerned crossing had degraded significantly, repair maintenance in terms of grinding and welding was performed to extend its service life and avoid sudden failure. The repair procedure was as follows: first, the crossing rails were ground to remove surface damage (e.g., cracks, uneven wear and plastic deformation); second, the hollow regions caused by grinding were filled by welding; finally, the crossing rails were ground again to the desired profiles. Figure 11 shows the repaired crossing profile. Because of the current incapability of operating a train-borne grinding machine at a crossing, the repair was performed manually. The repaired profile depends on the experience of the technicians and may differ significantly from the nominal profile; this is the unknown degradation status for the trial detection.

### 4.2. Trial Evaluation

**Figure 12:**
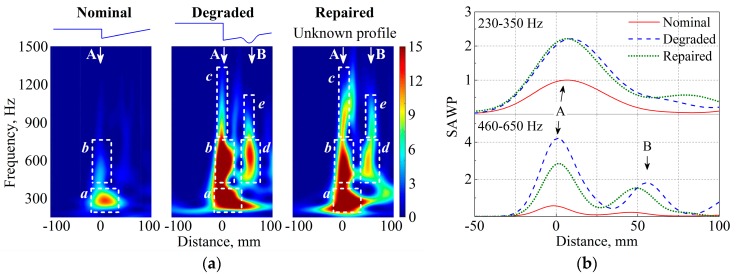
Comparison of ABA of nominal, degraded and repaired states. (**a**) WPS and (**b**) SAWP. The rectangles indicate the major characteristics. Arrows A and B indicate the location of degradation types 1 and 2.

Figure 12a compares the WPS of the measured ABA, and Figure 12b compares the SAWP, namely, ${\bar{W}}_{Nom}^{2}$ of the nominal state, ${\bar{W}}_{Deg}^{2}$ of the degraded state and ${\bar{W}}_{Rep}^{2}$ of the repaired states. In the nominal state, there is only one position with the value of ${\bar{W}}^{2}\left[460,650\right]$ exceeding 0.3; there are two positions in both the degraded and repaired states: the first peak is related to the intrinsic geometric discontinuity, while the second is related to the local irregularity of the crossing nose (i.e., degradation type 2). This information indicates that the repair did not completely remove the local irregularity of the crossing nose. The value of ${\bar{W}}_{Rep}^{2}\left[460,650\right]$ is 15% smaller than the value of ${\bar{W}}_{Deg}^{2}\left[460,650\right]$, indicating that the severity of the local irregularity was reduced.

At arrow A, the value of ${\bar{W}}_{Rep}^{2}\left[460,650\right]$ decreased by 31% after repair, whereas the value of ${\bar{W}}_{Rep}^{2}\left[230,350\right]$ remained at a similar level as that in the degraded state. Because the energy concentration at arrow A corresponds to the severity of the uneven deformation between the wing rail and the crossing nose (i.e., degradation type 1), it is determined that the repair failed to compensate for the uneven deformation between the wing rail and the crossing nose. In summary, Figure 12 indicates that the repair reduced the severity of the local irregularity of the crossing nose (degradation type 2), but failed to compensate for the uneven deformation between the wing rail and the crossing nose (degradation type 1).

### 4.3. Verification

**Figure 13:**
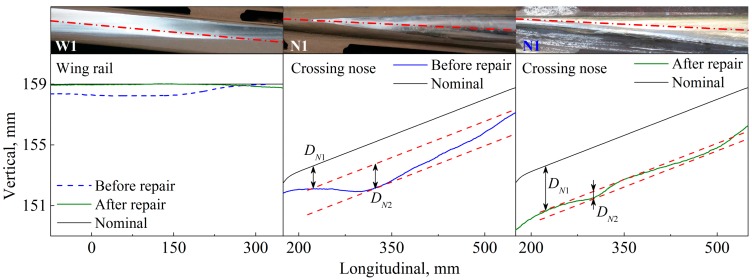
Change of the crossing profile due to repair. $D_{N1}$ and $D_{N2}$ indicate the height difference of the crossing nose.

To examine the evaluation results, a 3D profile measurement was performed on the repaired crossing. Figure 13 compares the measured profile along W1 and N1. The height of the repaired wing rail was nearly restored to the nominal state, whereas the height of the repaired crossing nose became even lower than that in the degraded state. Consequently, the uneven deformation between the wing rail and the crossing nose was exacerbated by the repair. The local irregularity $D_{N2}$ of the crossing nose (degradation type 2) still existed after the repair but was not as severe as the degraded state. Therefore, the evaluation results made by the ABA measurement are considered reliable.

In this study, a detection algorithm is developed and verified relying on in situ ABA and 3D profile measurements taken at a single track site. At the site, two crossings with three different health conditions were measured: the first measurement occurred on a degraded crossing, the second measurement was performed shortly after repair, and the third measurement was performed on a new crossing at the same site after the repaired crossing was replaced because of severe cracking.

The advantage of focusing on a single site is that the disturbance of non-uniform track conditions on the characteristic vibrations of ABA can be minimized. It would be highly beneficial to demonstrate the detection algorithm on additional crossings. However, access to additional track sites is strictly limited by the safety regulations of the Dutch railway network authority. In this study, we were allowed to perform measurements at only one site, and it required 1.5 years to collect information for three different health conditions. For this feasibility study, the experiment was satisfactorily conducted and provided new information that is helpful for developing a better maintenance strategy. Signal processing together with physical insight into the system provided reliable information for verifying the quality of maintenance actions.

## 5. Discussion: Aspects Considered Helpful for Extending the ABA System to Other Examples

To extend the ABA system to other examples, the following aspects should be taken into account:

- The effect of non-identical wheel-rail trajectory on the characteristic frequencies of ABA. In the real-life implementation of the ABA system, it is impossible to keep the identical wheel-rail trajectory among measurements. On one hand, it is difficult to keep in-service trains with a controlled constant train speed; on the other, the wheel-rail trajectory is affected by the randomness of vehicle-track interaction (e.g., hunting oscillation). The effect of non-identical trajectory on the characteristic frequencies of ABA must be analyzed. In the literature, it is found that the characteristic frequencies of ABA are related to the natural response of the vehicle-track system [38], so that the characteristic frequencies of ABA, and thus the capability of the proposed detection algorithm, are not greatly affected by non-identical wheel-rail trajectory.
- The effect of the crossing type on the characteristic frequencies of ABA. In this study, the proposed detection algorithm is demonstrated and verified on the crossing type of 54E1-1:9. Because the natural response of crossings may differ from one type to another, the characteristic frequencies of ABA on other crossing types should be extracted. To overcome the limitations on field track testing, computer-aided approaches (e.g., finite element simulation [24] and machine learning [42]) can be used for virtual testing. The ABA system can be more conveniently extended to various crossing types using more flexible and relatively faster numerical modeling rather than time-consuming and expensive in situ measurements.

Moreover, by the correct correlation of the gathered data from different sensors, the proposed detection method can be extended to other track components (e.g., switch and catenary). For example, it is possible to estimate the location and severity of the catenary degradation by collecting and correlating the data of the pantograph-catenary contact force [43] and the catenary irregularities [44] instead of ABA and crossing profiles. In addition to monitoring railway infrastructure, the proposed method can be extended to a broader range of applications, such as inspecting the defects of bearings [45].

## 6. Conclusions and Further Work

In this study, the capability of the ABA system to detect crossing degradation was investigated. For this purpose, in situ ABA and 3D profile measurements were conducted on both nominal and degraded crossings to extract characteristic vibrations related to the degradation. ABA measurements were then performed on a crossing with an unknown degradation status condition as a trial detection, and the capability of the method was verified using 3D profile measurements and field observations. The following conclusions are drawn:

(1) The ABA system can identify two types of crossing degradation. The first type is uneven deformation between the wing rail and the crossing nose, and the second type is local irregularity in the longitudinal slope of the crossing nose.
(2) Deformation of the crossing nose that is more severe than that of the wing rail exacerbates wheel-rail impact during the facing motion of vehicles, increasing the energy concentrated at the characteristic frequencies of 230–350 and 460–650 Hz. The severity of the uneven deformation can be evaluated by the energy concentration at these frequencies.
(3) The presence of a local irregularity at the crossing nose increases the vibration energy at the characteristic frequencies of 460–650 Hz. The location of the irregularity can be determined by the spatial distribution of these frequencies, while the severity can be evaluated by the energy concentration at these frequencies.
(4) The ABA system can detect crossing degradation at measuring speeds as low as 26–28 km/h. Therefore, the capability of the method in large-scale networks is not restricted to the low operational speed often specified at crossings (40–80 km/h on the Dutch railway).

Planned future work includes investigating the characteristic vibrations of the degradation at various crossing types and over a broad range of measuring speeds. Possible future studies also include integrating the ABA system with other nondestructive testing technologies (e.g., those capable of crack detection) such that infrastructure managers can be provided with more comprehensive information for decision making on maintenance work. In addition, a numerical model will be developed for virtual testing to analyze the characteristic signals of various degradation types.

## Supplementary Materials

Supplementary File 1

## Nomenclature

The following abbreviations and symbols are used in this manuscript:

ABA	axle box acceleration
CWT	continuous wavelet transform
GPS	global positioning system
GWPS	global wavelet power spectra
SAWP	scale-averaged wavelet power
WPS	wavelet power spectrum
$C_{\delta }$	reconstruction factor
$D_{N1}$, $D_{N2}$	height difference of crossing nose between nominal and degraded states
$D_{W}$	height difference of wing rail between nominal and degraded states
$f_{1}$, $f_{2}$	frequency
N	number of points in time series
n	continuous variable for translation
s	wavelet scale
$W_{n}$	wavelet coefficients
$\left|W_{n}^{2}\right|$	wavelet power spectrum
${\bar{W}}_{n}^{2}\left[f_{1},f_{2}\right]$	scale-averaged wavelet power of frequency band $f_{1}$ to $f_{2}$
x	analyzed ABA signal
γ	scale-averaged wavelet power at nominal state
$\delta_{j}$	scale step of scale-averaged wavelet power
$\delta_{t}$	time step
ψ	mother wavelet
∗	complex conjugate
